# Implementation of digital HIV services in Guangzhou’s primary health-care system, China

**DOI:** 10.2471/BLT.24.291921

**Published:** 2024-12-03

**Authors:** Jun Wang, Yu-Zhou Gu, Yong-Heng Lu, Ju-Shuang Li, Ye-Fei Luo, Yan-Shan Cai, Zhi-Gang Han, Chun Hao

**Affiliations:** a School of Public Health, Sun Yat-Sen University, 74 Zhongshan No. 2 Rd, Yuexiu District, Guangzhou 510080, Guangdong, China.; b Guangzhou Center for Disease Control and Prevention, Guangzhou, Guangdong, China.; c Guangzhou Lingnan Community Support Center, Guangzhou, Guangdong, China.

## Abstract

**Objective:**

To describe changes in access to voluntary human immunodeficiency virus (HIV) counselling and testing services following the implementation of a mini-application (app) at primary health-care facilities across Guangzhou, China.

**Methods:**

In 2018, the Guangzhou Center for Disease Control and Prevention and the Lingnan Community Support Center co-developed WellTest, a mini-app within the WeChat environment, to address voluntary counselling and testing service needs. The mini-app provides on-demand information for clients, links them to health services, and allows users to provide feedback on health-care quality and share test results with partners. We retrieved data from the National HIV/AIDS Prevention and Control Information System and the app backend to assess adoption and service use after implementation of WellTest in primary health-care facilities in December 2018.

**Findings:**

Between 2018 and 2023, WellTest adoption at primary health-care facilities increased from 1.5% (2/134) to 90.1% (172/191). The annual number of visits for voluntary HIV counselling and testing services at these facilities rose from 7376 to 20 002, representing 83.6% of all 23 930 voluntary HIV counselling and testing visits in 2023, compared with 36.6% (7376/20 129) in 2018. The proportion of newly diagnosed HIV infections at primary health-care facilities increased from 21.7% (187/862) to 50.4% (239/474).

**Conclusion:**

The increased access to HIV services in Guangzhou’s primary health-care facilities highlights the value of digital health interventions in addressing service delivery gaps and meeting the needs of providers and clients. Expanding such interventions has the potential to strengthen decentralized health care and disease screening in low-resource settings.

## Résumé

**Objectif:**

Décrire les changements dans l’accès aux services volontaires de conseil et de dépistage du virus de l’immunodéficience humaine (VIH) après la mise en œuvre d’une mini-application dans les établissements de soins de santé primaires de l’ensemble de Canton en Chine.

**Méthodes:**

En 2018, le Centre de contrôle et de prévention des maladies de Canton et le Centre de soutien communautaire de Lingnan ont mis conjointement au point WellTest, une mini-application au sein de l’environnement WeChat, afin de répondre aux besoins en matière de services volontaires de conseil et de dépistage. Cette mini-application fournit des informations à la demande des clients, les relie aux services de santé et permet aux utilisateurs de donner leur avis sur la qualité des soins de santé et de partager les résultats de dépistage avec leurs partenaires. Nous avons récupéré des données du Système national d’information sur la prévention et le contrôle du VIH/sida et du backend de l’application afin d’évaluer l’adoption et l’utilisation des services après la mise en œuvre de WellTest dans les établissements de soins de santé primaires en décembre 2018.

**Résultats:**

Entre 2018 et 2023, l’adoption de WellTest dans les établissements de soins de santé primaires est passée de 1,5% (2 sur 134) à 90,1% (172 sur 191). Le nombre annuel de visites pour des services volontaires de conseil et de dépistage du VIH dans ces établissements est passé de 7376 à 20 002, soit 83,6% de l’ensemble des 23 930 visites volontaires de conseil et de dépistage du VIH en 2023, contre 36,6% (7376 sur 20 129) en 2018. La proportion d’infections au VIH nouvellement diagnostiquées dans les établissements de soins de santé primaires est passée de 21,7% (187 sur 862) à 50,4% (239 sur 474).

**Conclusion:**

L’accès accru aux services de lutte contre le VIH dans les établissements de soins de santé primaires de Canton met en évidence la valeur des interventions de santé numérique destinées à combler les lacunes dans la prestation de services et à répondre aux besoins des prestataires et des clients. L’extension de ces interventions permettrait de renforcer les soins de santé décentralisés et le dépistage des maladies dans les environnements à faibles ressources.

## Resumen

**Objetivo:**

Describir los cambios en el acceso a los servicios de asesoramiento y pruebas voluntarias del virus de la inmunodeficiencia humana (VIH) tras la implementación de una miniaplicación en los centros de atención primaria de Guangzhou en China.

**Métodos:**

En 2018, el Centro para el Control y la Prevención de Enfermedades de Guangzhou y el Centro de Apoyo Comunitario de Lingnan desarrollaron conjuntamente WellTest, una miniaplicación dentro del entorno WeChat, para abordar las necesidades de servicios de asesoramiento y pruebas voluntarias. La miniaplicación proporciona información a los usuarios, los pone en contacto con los servicios sanitarios y les permite dar su opinión sobre la calidad de la atención sanitaria y compartir los resultados de las pruebas con sus socios. Se recuperaron datos del Sistema nacional de información sobre la prevención y el control del VIH/sida y del backend de la aplicación para evaluar la adopción y el uso del servicio tras la implementación de WellTest en los centros de atención primaria en diciembre de 2018.

**Resultados:**

Entre 2018 y 2023, la adopción de WellTest en los centros de atención primaria aumentó del 1,5% (2/134) al 90,1% (172/191). El número anual de visitas para los servicios de asesoramiento y pruebas voluntarias del VIH en estas instalaciones aumentó de 7376 a 20 002, lo que representa el 83,6% de todas las 23 930 visitas de asesoramiento y pruebas voluntarias del VIH en 2023, en comparación con el 36,6% (7376/20 129) en 2018. El porcentaje de nuevas infecciones por VIH diagnosticadas en los centros de atención primaria aumentó del 21,7% (187/862) al 50,4% (239/474).

**Conclusión:**

El mayor acceso a los servicios relacionados con el VIH en los centros de atención primaria de Guangzhou evidencia el valor de las intervenciones sanitarias digitales para abordar las deficiencias en la prestación de servicios y satisfacer las necesidades de proveedores y clientes. La ampliación de este tipo de intervenciones puede reforzar la atención sanitaria descentralizada y la detección de enfermedades en entornos con pocos recursos.

## ملخص

**الغرض:**

وصف التغييرات في الوصول إلى الخدمات الطوعية للاستشارات والاختبار لفيروس نقص المناعة البشرية (HIV)، بعد نشر التطبيق الصغير (app) في مرافق الرعاية الصحية الأولية في جميع أنحاء قوانغتشو، الصين.

**الطريقة:**

في عام 2018، قام مركز قوانغتشو لمكافحة الأمراض والوقاية منها، ومركز دعم المجتمع في لينغنان بتطوير WellTest، وهو تطبيق صغير داخل بيئة WeChat، لتلبية احتياجات الخدمات الطوعية للاستشارات والاختبار. يوفر التطبيق الصغير للعملاء معلومات عند الطلب، ويربطهم بالخدمات الصحية، ويسمح للمستخدمين بتقديم ملاحظات حول جودة الرعاية الصحية، ومشاركة نتائج الاختبار مع الشركاء. لقد قمنا باسترجاع البيانات من النظام الوطني لمعلومات الوقاية والمكافحة لفيروس نقص المناعة البشرية/الإيدز، والواجهة الخلفية للتطبيق لتقييم التبني واستخدام الخدمة بعد نشر WellTest في مرافق الرعاية الصحية الأولية في ديسمبر/كانون أول 2018.

**النتائج:**

بين عامي 2018 و2023، زاد تبني WellTest في مرافق الرعاية الصحية الأولية من %1.5 (2/134) إلى %90.1 (172/191). ارتفع العدد السنوي للزيارات للخدمات الطوعية للاستشارات والاختبار لفيروس نقص المناعة البشرية في هذه المرافق من 7376 إلى 20002، وهو ما يمثل %83.6 من إجمالي 23930 زيارة طوعية للاستشارات والاختبار لفيروس نقص المناعة البشرية في عام 2023، مقارنة بـ %36.6 (7376/20129) في عام 2018. ارتفعت نسبة الإصابات الجديدة بفيروس نقص المناعة البشرية التي تم تشخيصها حديثًا في مرافق الرعاية الصحية الأولية من %21.7 (187/862) إلى %50.4 (239/474).

**الاستنتاج:**

إن الوصول الزائد إلى خدمات فيروس نقص المناعة البشرية في مرافق الرعاية الصحية الأولية في قوانغتشو، يسلط الضوء على قيمة التدخلات الصحية الرقمية في معالجة فجوات تقديم الخدمات، وتلبية احتياجات مقدمي الخدمات والعملاء. إن توسيع مثل هذه التدخلات لديه إمكانية دعم الرعاية الصحية اللامركزية، وفحص الأمراض في البيئات ذات الموارد المنخفضة.

## 摘要

**目的:**

旨在描述在中国广州推行小程序（应用程序）后，基层卫生服务机构艾滋病咨询检测服务使用比例的变化。

**方法:**

2018年，广州市疾病预防控制中心和岭南伙伴社区支持中心共同开发了微信小程序查呗 (WellTest)，以解决艾滋病咨询检测服务需求。该小程序按用户需求提供信息和链接卫生服务，并允许用户就卫生服务质量提供反馈意见以及与伴侣分享检测结果。基于国家艾滋病防治工作信息系统及该小程序的后台数据，评估自2018年12月在基层卫生服务机构推行查呗后该程序的采用率和相关服务的使用情况。

**结果:**

在2018年至2023年期间，查呗 (WellTest)在基层卫生服务机构的采用率从1.5% (2/134) 上升至了90.1% (172/191)。这些机构的艾滋病自愿咨询检测服务年度访问次数从2018年的7,376次【占36.6% (7376/20,129)】增至了2023年的20,002次【占83.6%，(20,002/23,930)】。基层卫生服务机构检出的艾滋病毒新发感染者比例从21.7% (187/862)上升至了50.4% (239/474)。

**结论:**

广州基层卫生服务机构的艾滋病自愿咨询检测服务使用比例增加，突显了数字健康干预措施在解决服务供给缺口和满足服务提供者和客户需求方面的重要性。广泛推行此类干预措施有利于在资源匮乏且卫生保健和疾病筛查服务较分散的地区提高服务利用率。

## Резюме

**Цель:**

Представить описание изменений в доступе к услугам по добровольному консультированию и тестированию на вирус иммунодефицита человека (ВИЧ) после внедрения мини-приложения в учреждениях первичной медико-санитарной помощи в Гуанчжоу, Китай.

**Методы:**

В 2018 году Центр по контролю и профилактике заболеваний Гуанчжоу и Центр общественной поддержки Линьнан совместно разработали мини-приложение WellTest в среде WeChat для удовлетворения потребностей в сфере услуг добровольного консультирования и тестирования. Мини-приложение предоставляет клиентам информацию по запросу, связывает их с медицинскими службами, позволяет им оставлять отзывы о качестве медицинского обслуживания и делиться результатами анализов с партнерами. Для оценки внедрения и использования сервиса после внедрения WellTest в учреждениях первичной медико-санитарной помощи в декабре 2018 года были получены данные от Национальной информационной системы по профилактике и контролю ВИЧ/СПИДа и сервера приложения.

**Результаты:**

В период с 2018 по 2023 год количество случаев применения WellTest в учреждениях первичной медико-санитарной помощи увеличилось с 1,5% (2/134) до 90,1% (172/191). Ежегодное число посещений для добровольного консультирования и тестирования на ВИЧ в этих учреждениях выросло с 7376 до 20 002, что составляет 83,6% от всех 23 930 посещений для добровольного консультирования и тестирования на ВИЧ в 2023 году по сравнению с 36,6% (7376/20 129) в 2018 году. Доля впервые выявленных случаев ВИЧ-инфекции в учреждениях первичной медико-санитарной помощи увеличилась с 21,7% (187/862) до 50,4% (239/474).

**Вывод:**

Расширение доступа к услугам по профилактике и лечению ВИЧ-инфекции в учреждениях первичной медико-санитарной помощи Гуанчжоу подчеркивает ценность мероприятий в области цифрового здравоохранения для устранения недостатков в оказании услуг и удовлетворения потребностей поставщиков и клиентов. Расширение таких мероприятий может способствовать укреплению децентрализованного здравоохранения и скрининга заболеваний в условиях ограниченных ресурсов.

## Introduction

Human immunodeficiency virus (HIV) testing is a key component in achieving the Joint United Nations Programme on HIV/AIDS target of ensuring that 95% of people living with HIV are aware of their status by 2025.^1^ Facility-based HIV voluntary counselling and testing services provide an important testing option of promptly linking individuals diagnosed with HIV to medical care, treatment and support services.^2^^,^^3^

In China, conventional providers of voluntary counselling and testing services are Center for Disease Control and Prevention (CDC) facilities at the city or district level. However, shortages in human resources and dispersed coverage hinder access to convenient and high-quality services.^4^ To address this accessibility issue, a national strategy, published in 2009, aims to shift voluntary counselling and testing services to primary health-care settings.^5^ Despite this strategy, the use of these services in primary health-care facilities remained low due to limited awareness, the concerns about increased HIV-related stigma in the community and concerns about service quality.^6^^–^^10^

In 2018, Guangdong province accounted for 18.4% (11 806/64 170) of all reported HIV infections in China.^11^^,^^12^ In the province, individuals faced difficulties obtaining information about available voluntary counselling and testing services, due to inconsistent listings of primary health-care facilities’ contact details on CDC websites. Most individuals had to visit facilities to register for testing; however, appointments were often fully booked on the day of the visit and these visits could also increase fears of stigmatization. Consequently, in Guangzhou the use of these services at primary health-care facilities remained low whereas CDC services were often overloaded. In 2018, 12 CDC facilities provided 61.3% (12 336/20 129) of the city’s total voluntary counselling and testing services, while 134 primary health-care facilities contributed to only 36.6% (7376/20 129) of these services (Chun Hao, Sun Yat-Sen University, Guangzhou, China, unpublished data, November 2024).

Furthermore, informing clients of HIV test results by phone or text was time-consuming for staff. Additionally, government-led service quality evaluations of primary health-care facilities lacked client involvement and relied on annual inspections or spot checks.

Digital health interventions offer opportunities to improve the primary health-care system by increasing access to care and information, reducing waiting times and supporting collaborative interprofessional practice.^13^^–^^18^ To improve the use of voluntary HIV counselling and testing services at primary health-care facilities in Guangzhou, we developed WellTest, a mini-application (app) within the WeChat ecosystem (Tencent Holdings Ltd, Shenzhen, China), for these services. Here we present the findings from implementing this mini-app at primary health-care facilities.

## Methods

### Setting

Guangzhou, the capital city of Guangdong Province, has a population of around 20 million. In 2018, there were 134 primary health-care facilities, including community health centres, community health stations and township health centres in Guangzhou.^19^ Furthermore, Guangzhou had 12 CDCs (11 district-level and 1 city-level), each of which had a centre-based health-care facility. Public health services in each district are primarily provided by either primary or centre-based health-care facilities. All public health services are government funded and provided at no cost to users. 

CDC facilities provide services such as infectious and chronic disease prevention, emergency public health response, epidemic management, health hazard detection, laboratory testing, health education and promotion and technical guidance.^20^ The city-level CDC oversees district-level CDCs, which guide and supervise primary health-care facilities in providing services, including infectious and chronic disease management and control.^20^

Primary health-care facilities provide general clinical care and basic public health services^21^ and are located in every subdistrict or serve every 30 000 to 100 000 residents, ensuring that basic public health services are accessible for all.^22^ Since 2009, each primary health-care facility has had 1–2 trained voluntary HIV counselling and testing counsellors. 

Although both CDC and primary health-care facilities offer HIV voluntary counselling and testing services, clients have traditionally recognized CDC facilities as the authority on infectious disease control, and have predominantly sought HIV testing at CDC facilities. 

The nonprofit organization Lingnan Community Support Centre, in partnership with Guangzhou CDC, offers peer-friendly voluntary counselling and testing, and conducts about three fifths of HIV tests for men who have sex with men in Guangzhou. 

### The digital intervention

To increase the use of voluntary counselling and testing services among the general population, the Guangzhou CDC and the Lingnan Community Support Centre developed the mini-app WellTest in 2018. The mini-app originates from the Lingnan website, set up in 2010 to improve the appointment scheduling process for the centre.^23^ A mini-app functions similarly to standalone apps, but is integrated into the WeChat ecosystem. WeChat, a Chinese instant messaging, social media and mobile payment app, has over 1.33 billion users in China.^24^ The CDC reallocated HIV/AIDS prevention funds to support the multicentre development of WellTest. The development process included incorporation of facility information into the mini-app and providing access permission to health-care facilities.

**Fig. 1:**
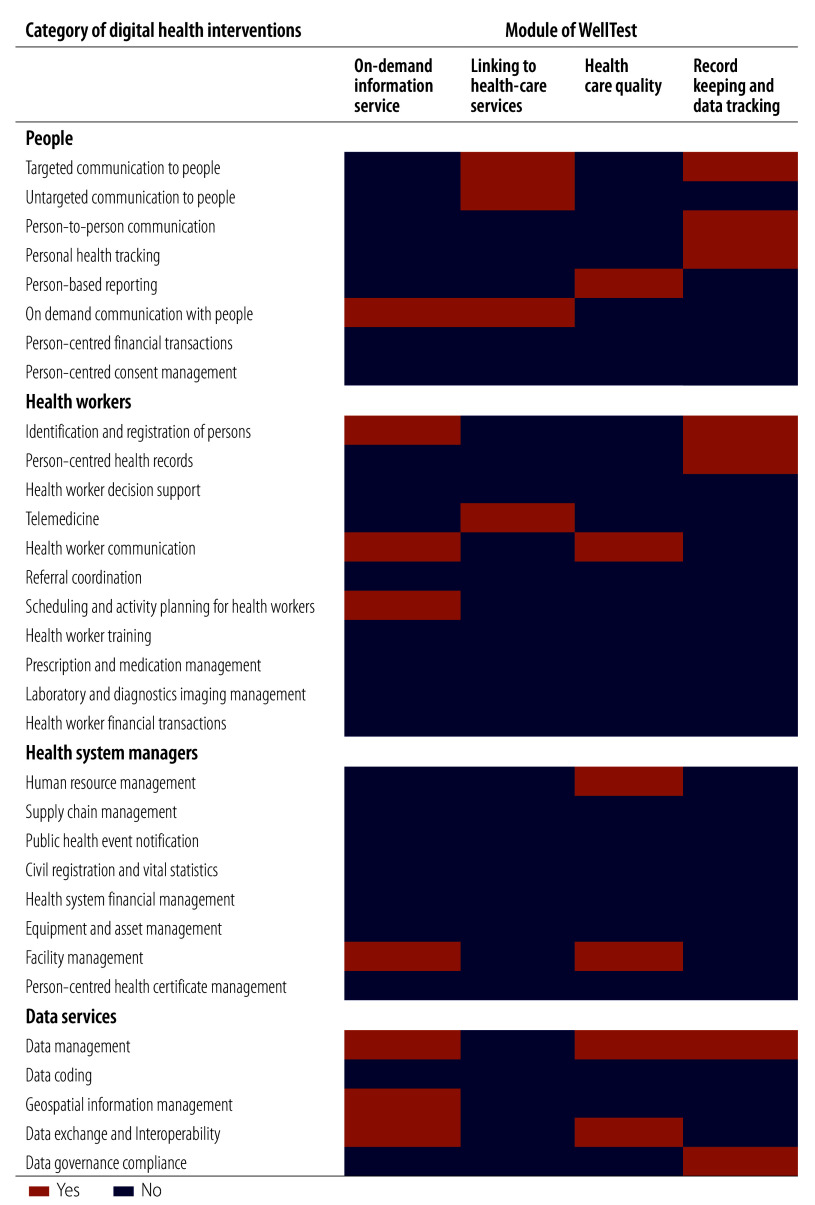
Modules of the mini-app WellTest and classifications of digital health interventions

WellTest is designed to address gaps in the voluntary counselling and testing services and consists of four modules: (i) on-demand information service; (ii) linking to health-care services; (iii) health care quality; and (iv) record keeping and data tracking. The modules feature various World Health Organization (WHO) categories of digital health interventions (Fig. 1).^33^

App: application.

Note: categories are based on *WHO Classification of digital interventions, services and applications in health*, 2nd ed.^33^

The on-demand information service module aims to increase awareness of in-person resources, such as a brief introduction, location and available schedules of HIV voluntary counselling and testing facilities, and streamline appointment processes. The module maps nearby facilities, allowing quick and convenient access to information about facilities offering free voluntary counselling and testing services. Clients can view appointment schedules and book appointments online after verifying their phone numbers, bypassing the general outpatient registration process. In-person appointments for HIV counselling and testing are then conducted at the designated time and location. The module also allows clients to cancel appointments.

The health service linking module offers personalized online assistance for HIV-related inquiries, connecting individuals with trained counsellors who provide confidential consultations, guidance and care recommendations. The goal is to direct at-risk individuals to suitable in-person HIV-related health-care services, including voluntary counselling and testing, self-testing kit collection, and pre- and post-exposure prophylaxis services at voluntary counselling and testing facilities.

In the health-care quality module, clients can provide anonymous feedback on their service experiences. This feedback includes both quantitative ratings and qualitative free-text entries, typically addressing clients’ perceptions of staff attitudes, professionalism, environment and other factors needed for comprehensive evaluations. The facility rating, available in the mini-app, aids clients in identifying and choosing high-quality facilities based on client-centric ratings.

The record-keeping and data-tracking module securely records the use of voluntary counselling and testing services and automatically stores HIV testing results on dedicated servers. The module integrates client data with the National HIV/AIDS Prevention and Control Information System, simplifying documentation for these services, by using clients’ personal information stored in WeChat. Following in-person testing at a facility, electronic reports of HIV test results, certified by Guangzhou CDC, are delivered via WellTest. Clients who provide consent can share their reports with partners via WeChat. Subsequently, recipients of shared electronic reports can schedule an HIV test by accessing the on-demand information module in WellTest via a link in the shared report. For friends seeking to know each other’s HIV status, each electronic report shared can serve as a reminder to get tested or share test results. The automated and structured electronic record system reduces staff workload by abolishing manual data entry, and enables efficient performance assessments. 

WellTest uses dedicated servers with robust encryption, restricted access and compliance measures to store client data. Only authorized counselling and testing personnel can export data via a designated account, with sensitive personally identifiable information securely encrypted. Access to unencrypted data requires mobile phone verification, and all exported data are strictly protected by national privacy laws to prevent leaks. Data security is further reinforced by partnership with the privacy-focused WeChat platform.

### Implementation of WellTest

**Fig. 2:**
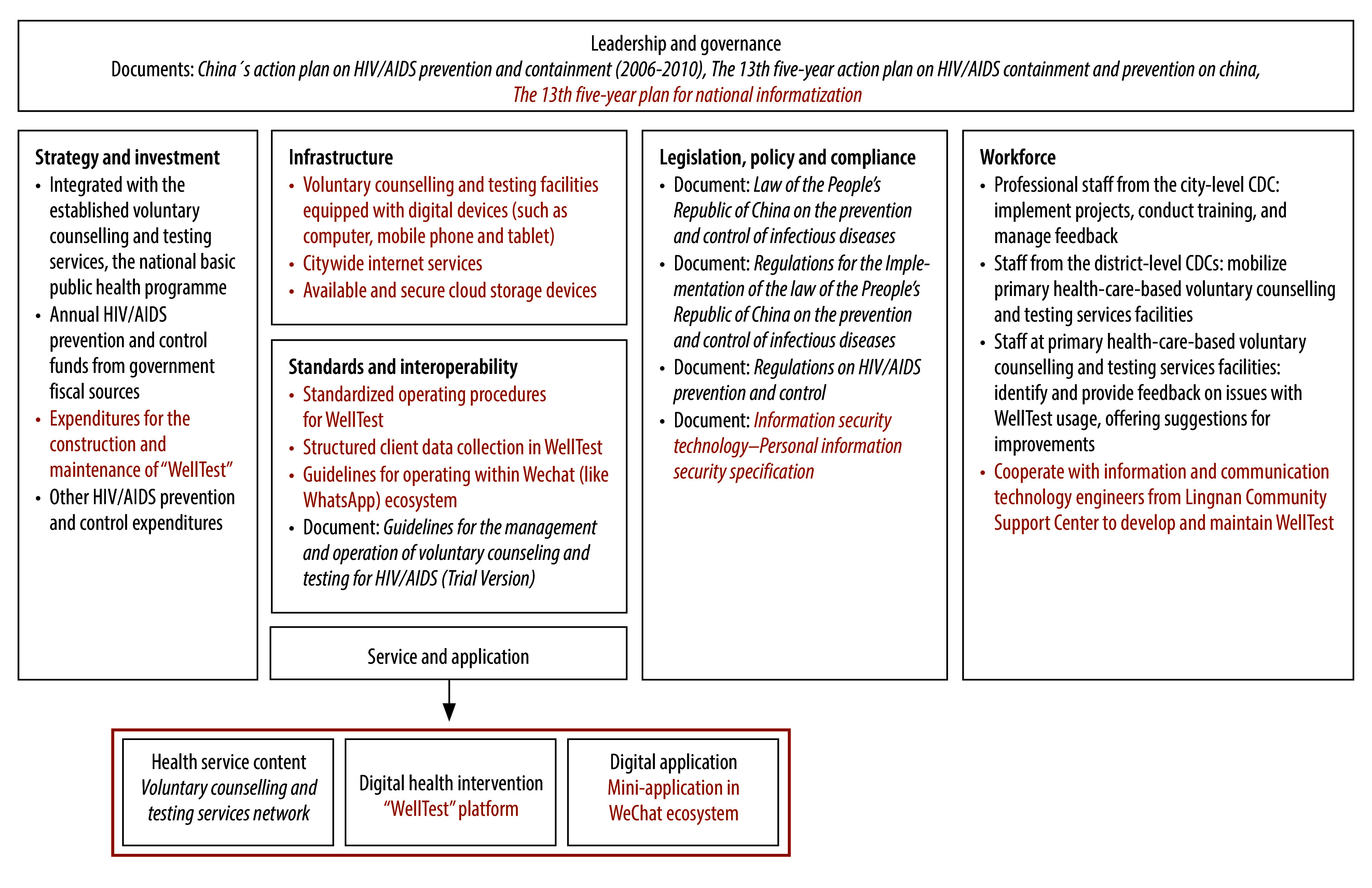
Components contributing to WellTest implementation^25^^–^^32^

AIDS: acquired immune deficiency syndrome; CDC: Center for Disease Control and Prevention; HIV: human immunodeficiency virus.

Note: Text in orange highlights items related to the implementation of WellTest.

Aligning with the *WHO guideline: recommendations on digital interventions for health system strengthening*,^34^ Fig. 2 outlines the seven components contributing to the implementation of WellTest, that is, (i) leadership and governance; (ii) strategy and investment; (iii) infrastructure; (iv) standards and interoperability; (v) service and application; (vi) legislation, policy and compliance; and (vii) workforce.

HIV: human immunodeficiency virus.

#### Facilities

In the first stage of implementation, Guangzhou CDC coordinated with district-level CDC officials to assess the demand and suitability of WellTest for primary health-care facilities, considering existing voluntary counselling and testing services and staffing levels.

Guangzhou CDC’s HIV Prevention and Control Department led the implementation, with district-level CDCs coordinating staff members at primary health-care facilities. The implementation began with an initial roll-out in CDC-based facilities before expanding to primary health-care facilities. Each district-level CDC chose between two implementation methods: either piloting in proactive facilities followed by broader roll-out or full roll-out in all facilities. To digitalize the appointment process, user registration, notification of test results, and to integrate facilities into the WellTest network, the developers collected service-related information and operational management information from facilities. For staff members, Guangzhou CDC’s HIV Prevention and Control Department provided a half-day in-person training on the standard operating procedure. The implementation included a workshop to train voluntary counselling and testing counsellors on using the mini-app. After the workshop, counsellors could choose to adopt WellTest. Primary health-care facilities established after the roll-out could implement WellTest if they followed the same training and integration steps. 

City- and district-level CDC staff members provided ongoing support to primary health-care facilities using WellTest via a WeChat group, where facilities can provide feedback and request assistance for any issue.

To improve future implementation, the Guangzhou CDC and district-level CDCs held a symposium with personnel involved in the implementation of WellTest to review and analyse the barriers and facilitators in the implementation process. 

#### Awareness raising

To increase awareness, CDCs and community organizations promoted WellTest through their websites and WeChat official accounts by displaying a QR (quick response) code, and doing outreach activities. Additionally, strategies included (i) having counsellors who use WellTest in facilities introduce the mini-app to clients; (ii) partnering with health-care departments to display WellTest advertisements on machines distributing free contraceptives; (iii) embedding WellTest information in promotional materials, such as brochures, posters and videos, and advertising on public transport billboards around World AIDS Day; and (iv) distributing WellTest-branded products like tote bags, baseball caps, mugs and badges for free to facility members, allowing them to promote the mini-app.

### Data sources and variables

We used data from the National HIV/AIDS Prevention and Control Information System and the WellTest platform backend. From the national system, we extracted clients’ age, gender as given on their identification card, marital status, education level, test results, reasons for seeking services, HIV-testing history and service provider. The WellTest backend automatically tracks annual online consultations, service quality reviews, electronic report sharing and views.

The cost and core staffing needed for the development and operation of the WellTest platform were jointly assessed by the Guangzhou CDC and the Lingnan Community Support Center.

### Ethical considerations

The HIV voluntary counselling and testing services are part of national basic public health services, and data provided by the HIV Prevention and Control Department of Guangzhou CDC were anonymized. We analysed these data with approval from Guangzhou CDC and no additional ethical review was required.

## Results

Following the implementation of WellTest in primary health-care facilities providing voluntary counselling and testing to the population in Guangzhou in December 2018, the proportion of adopting facilities increased from 1.5% (2/134) in 2018 to 90.1% (172/191) in 2023. Among the facilities that have implemented WellTest, 87.8% (151/172) were community health centres and 55.2% (95/172) are located in central urban areas (Table 1).^35^

**Table 1:** Adoption of the mini-app WellTest in primary health-care facilities providing HIV voluntary counselling and testing services, Guangzhou, China, 2018–2023

Type and location	No. (%)																
	2018 (*n* = 134)			2019 (*n* = 179)			2020 (*n* = 180)			2021 (*n* = 185)			2022 (*n* = 186)			2023 (*n* = 191)	
	Yes	No		Yes	No		Yes	No		Yes	No		Yes	No		Yes	No
**Total**	**2**	**132**		**86**	**93**		**120**	**60**		**150**	**35**		**150**	**36**		**172**	**19**
**Type of primary health-care facility**																	
Community health stations	0 (0.0)	4 (3.0)		3 (3.5)	2 (2.2)		4 (3.3)	2 (3.3)		7 (4.7)	1 (2.9)		7 (4.7)	1 (2.8)		10 (5.8)	1 (5.3)
Community health centres	2 (100.0)	114 (86.4)		82 (95.3)	72 (77.4)		113 (94.2)	41 (68.3)		138 (92.0)	19 (54.3)		138 (92.0)	20 (55.6)		151 (87.8)	9 (47.4)
Township health centres	0 (0.0)	14 (10.6)		1 (1.2)	19 (20.4)		3 (2.5)	17 (28.3)		5 (3.3)	15 (42.9)		5 (3.3)	15 (41.7)		11 (6.4)	9 (47.4)
**Administrative districts**																	
Baiyun	0 (0.0)	1 (0.8)		0 (0.0)	19 (20.4)		10 (8.3)	9 (15.0)		22 (14.7)	0 (0.0)		22 (14.7)	0 (0.0)		23 (13.4)	0 (0.0)
Conghua	0 (0.0)	9 (6.8)		0 (0.0)	11 (11.8)		0 (0.0)	11 (18.3)		0 (0.0)	11 (31.4)		0 (0.0)	11 (30.6)		0 (0.0)	11 (57.9)
Haizhu	0 (0.0)	23 (17.4)		25 (29.1)	0 (0.0)		26 (21.7)	0 (0.0)		26 (17.3)	0 (0.0)		26 (17.3)	0 (0.0)		26 (15.1)	0 (0.0)
Huadu	0 (0.0)	17 (12.9)		0 (0.0)	17 (18.3)		0 (0.0)	17 (28.3)		0 (0.0)	17 (48.6)		0 (0.0)	18 (50.0)		15 (8.7)	3 (15.8)
Huangpu	0 (0.0)	16 (12.1)		14 (16.3)	2 (2.2)		14 (11.7)	2 (3.3)		14 (9.3)	2 (5.7)		14 (9.3)	2 (5.6)		16 (9.3)	1 (5.3)
Liwan	1 (50.0)	0 (0.0)		1 (1.2)	18 (19.4)		19 (15.8)	0 (0.0)		19 (12.7)	0 (0.0)		19 (12.7)	0 (0.0)		19 (11.0)	0 (0.0)
Nansha	1 (50.0)	7 (5.3)		8 (9.3)	0 (0.0)		8 (6.7)	0 (0.0)		8 (5.3)	0 (0.0)		8 (5.3)	0 (0.0)		8 (4.7)	0 (0.0)
Panyu	0 (0.0)	12 (9.1)		0 (0.0)	16 (17.2)		0 (0.0)	16 (26.7)		14 (9.3)	3 (8.6)		14 (9.3)	3 (8.3)		15 (8.7)	2 (10.5)
Tianhe	0 (0.0)	28 (21.2)		22 (25.6)	7 (7.5)		26 (21.7)	3 (5.0)		29 (19.3)	1 (2.9)		29 (19.3)	1 (2.8)		32 (18.6)	1 (5.3)
Yuexiu	0 (0.0)	18 (13.6)		16 (18.6)	2 (2.2)		17 (14.2)	1 (1.7)		18 (12.0)	0 (0.0)		18 (12.0)	0 (0.0)		18 (10.5)	0 (0.0)
Zengcheng	0 (0.0)	1 (0.8)		0 (0.0)	1 (1.1)		0 (0.0)	1 (1.7)		0 (0.0)	1 (2.9)		0 (0.0)	1 (2.8)		0 (0.0)	1 (5.3)

HIV: human immunodeficiency virus.

**Fig. 3:**
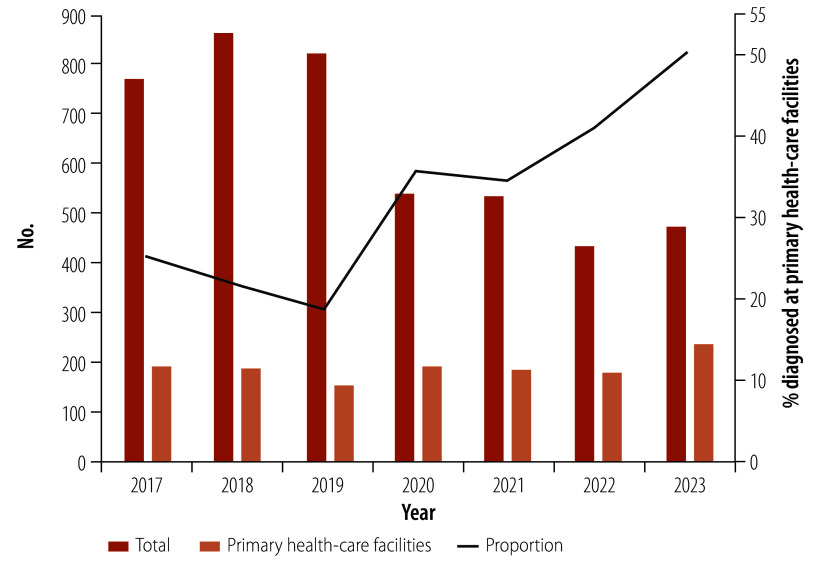
People newly diagnosed with HIV at primary health-care facilities, Guangzhou, China, 2017–2023

Between 2018 and 2023, WellTest served over 400 000 clients, with the annual number of visits for voluntary counselling and testing services facilitated by WellTest surging from 21 in 2018 to 18 313 in 2023. The annual number of individuals receiving an HIV diagnosis at primary health-care facilities also increased from 187 in 2018 to 239 in 2023 (Table 2). Notably, the proportion of individuals diagnosed at a primary health-care facility, relative to all newly diagnosed individuals with HIV increased from 21.7% (187/862) to 50.4% (239/474; Fig. 3). During this period, 59 846 HIV-related online consultations had been provided to clients.

**Table 2:** Characteristics of clients using voluntary counselling and testing services, Guangzhou, China, 2017–2023

Characteristic	No. of clients (%)^a^							
	Before implementation		Implementation					
	2017 (*n* = 19 759)		2018 (*n* = 20 129)	2019 (*n* = 21 849)	2020 (*n* = 14 128)	2021 (*n* = 17 827)	2022 (*n* = 17 491)	2023 (*n* = 23 930)
**Type of Facility**								
CDC-based facilities	13 050 (66.0)		12 336 (61.3)	9 937 (45.5)	4 195 (29.7)	4 141 (23.2)	3 456 (19.8)	3 593 (15.0)
Primary health-care facilities	6 333 (32.1)		7 376 (36.6)	11 317(51.8)	9 245 (65.4)	13 273 (74.5)	13 649 (78.0)	20 002 (83.6)
Others	376 (1.9)		417 (2.1)	595 (2.7)	688 (4.9)	413 (2.3)	386 (2.2)	335 (1.4)
**Newly diagnosed with HIV**	767		862	820	539	534	434	474
**Characteristics of clients at primary health-care facilities**								
Mean age, years (SD)	40.4 (17.1)		38.1 (16.3)	39.1 (17.5)	37.9 (16.5)	38.7 (17.0)	38.2 (16.3)	40.2 (17.4)
Gender^b^								
Male	4 044 (63.9)		4 579 (62.1)	6 732 (59.5)	5 520 (59.7)	7 926 (59.7)	8 623 (63.2)	11 769 (58.8)
Female	2 289 (36.1)		2 797 (37.9)	4 585 (40.5)	3 725 (40.3)	5 347 (40.3)	5 026 (36.8)	8 233 (41.2)
Marital status								
Married	3 645 (60.9)		4 192 (60.6)	5 898 (56.5)	4 554 (53.7)	6 655 (53.8)	6 248 (52.0)	10 000 (55.8)
Unmarried	2 156 (36.0)		2 531 (36.6)	4 198 (40.2)	3 651 (43.0)	5 361 (43.3)	5 428 (45.2)	7 397 (41.3)
Divorced or widowed	184 (3.1)		196 (2.8)	340 (3.3)	280 (3.3)	359 (2.9)	336 (2.8)	530 (3.0)
Unknown	348 (5.5)		457 (6.2)	881 (7.8)	760 (8.2)	898 (6.8)	1 637 (12.0)	2 075 (10.4)
Education level								
College or above	1 640 (27.4)		2 265 (32.7)	3 502 (33.6)	3 525 (41.5)	5 554 (44.7)	5 348 (44.3)	7 847 (43.8)
High school	1 857 (31.0)		1 937 (28.0)	2 763 (26.5)	1 981 (23.3)	2 710 (21.8)	2 935 (24.3)	3 861 (21.6)
Middle school	1 773 (29.6)		1 898 (27.4)	2 775 (26.6)	2 018 (23.8)	2 759 (22.2)	2 655 (22.0)	4 259 (23.8)
Primary school or below	722 (12.0)		824 (11.9)	1 390 (13.3)	966 (11.4)	1 392 (11.2)	1 139 (9.4)	1 949 (10.9)
Unknown	341 (5.4)		452 (6.1)	887 (7.8)	755 (8.2)	858 (6.5)	1 572 (11.5)	2 086 (10.4)
HIV test result								
Positive	193 (3.1)		187 (2.6)	153 (1.4)	193 (2.1)	184 (1.4)	179 (1.3)	239 (1.2)
Negative	5 993 (96.9)		7 013 (97.4)	10 958 (98.6)	9 016 (97.9)	13 033 (98.6)	13 388 (98.7)	19 712 (98.8)
Missing	147 (2.3)		176 (2.4)	206 (1.8)	36 (0.4)	56 (0.4)	82 (0.6)	51 (0.3)
Reason for using the service								
History of non-commercial, non-regular heterosexual behaviour	1 145 (18.1)		1 576 (21.4)	2 706 (23.9)	2 666 (28.8)	4 394 (33.1)	4 524 (33.1)	6 772 (33.9)
History of commercial heterosexual behaviour	686 (10.8)		823 (11.2)	2 215 (19.6)	1 242 (13.4)	1 756 (13.2)	1 602 (11.7)	2 003 (10.0)
History of men who have sex with men	1 173 (18.5)		1 228 (16.6)	1 146 (10.1)	1 324 (14.3)	1 629 (12.3)	1 664 (12.2)	2 183 (10.9)
History of medical- or blood-related procedures^c^	246 (3.9)		218 (3.0)	309 (2.7)	184 (2.0)	164 (1.2)	295 (2.2)	335 (1.7)
Spouse or regular sexual partner is HIV-positive	162 (2.6)		168 (2.3)	265 (2.3)	203 (2.2)	197 (1.5)	196 (1.4)	246 (1.2)
History of occupational exposure	36 (0.6)		104 (1.4)	94 (0.8)	54 (0.6)	106 (0.8)	158 (1.2)	94 (0.5)
History of injecting drugs	222 (3.5)		211 (2.9)	95 (0.8)	92 (1.0)	37 (0.3)	56 (0.4)	75 (0.4)
Mother is HIV-positive	14 (0.2)		13 (0.2)	22 (0.2)	8 (0.1)	11 (0.1)	10 (0.1)	3 (0.0)
Others	2 649 (41.8)		3 035 (41.1)	4 465 (39.5)	3 472 (37.6)	4 979 (37.5)	5 144 (37.7)	8 291 (41.5)
Ever tested for HIV								
Yes	1 801 (28.4)		1 935 (26.2)	2 142 (18.9)	2 477 (26.8)	3 703 (27.9)	3 896 (28.5)	6 060 (30.3)
No	4 532 (71.6)		5 441 (73.8)	9 175 (81.1)	6 768 (73.2)	9 570 (72.1)	9 753 (71.5)	13 942 (69.7)

CDC: Center for Disease Control and Prevention; HIV: human immunodeficiency virus; SD: standard deviation.

^a^ Number and percentages are presented in not otherwise stated.

^b^ Based on clients’ registration.

^c^ History of surgery, history of blood or blood products transfusion and history of donating plasma.

Note: for characteristics with missing information, the percentages are calculated using the sum of the known data as the denominators. Inconsistencies arise in some values due to rounding.

HIV: human immunodeficiency virus.

**Fig. 4:**
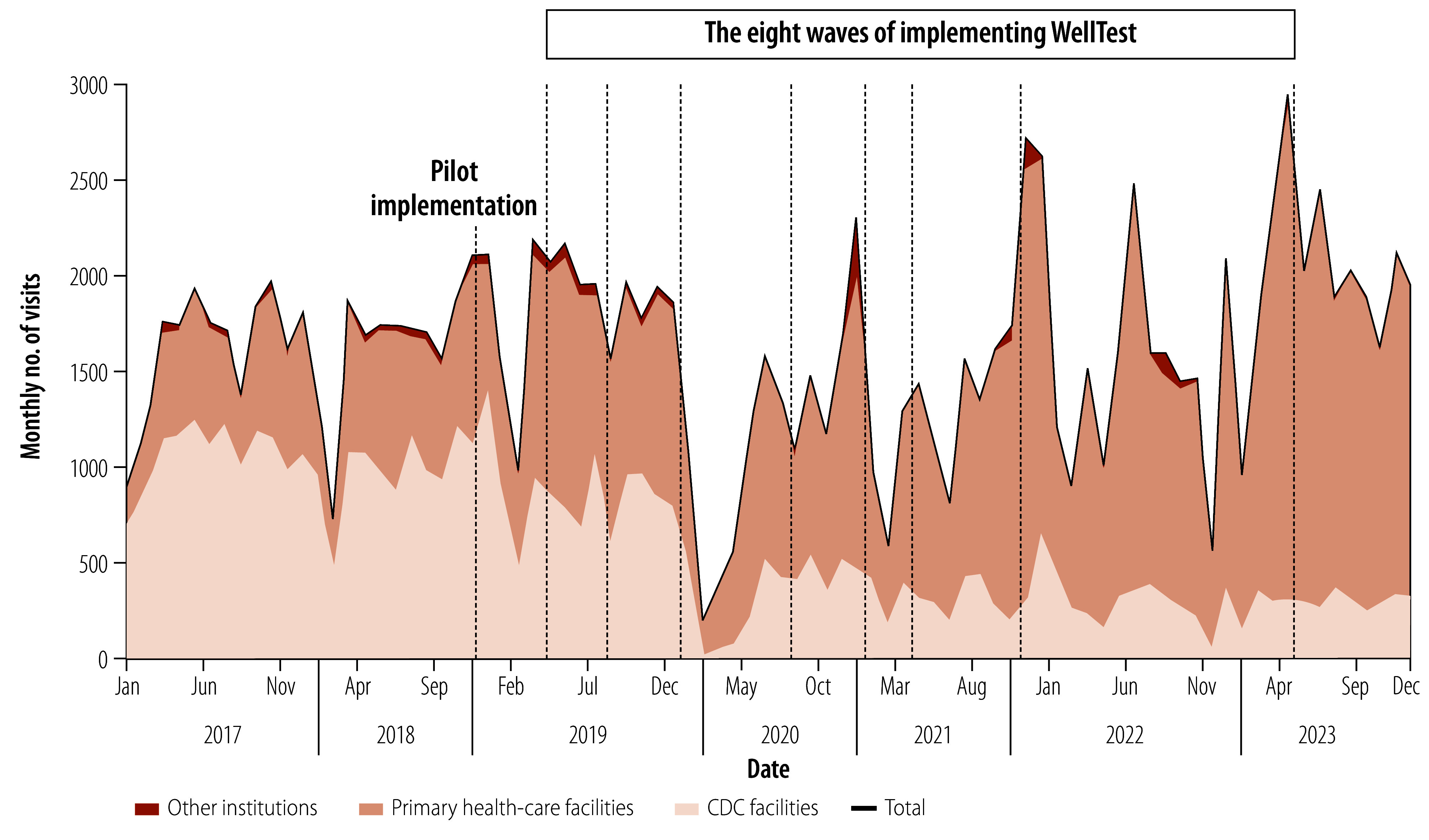
Monthly number of visits for voluntary counselling and testing services, by type of institution, Guangzhou, China, 2017–2023

In 2017, before the implementation of WellTest, the number of visits for voluntary counselling and testing services at primary health-care facilities was 6333, representing 32.1% of all 19 759 visits in Guangzhou. In 2023, this number had increased to 20 002 visits, constituting 83.6% of 23 930 visits that year (Table 2). Fig. 4 shows the monthly number of visits from various institution types. The monthly visits at primary health-care facilities almost doubled after the implementation of WellTest, from 592 in September 2018 to 1063 in November 2019.

CDC: Center for Disease Control and Prevention.

Between 2017 and 2023, clients using voluntary counselling and testing services at primary health-care facilities were predominantly males, and the mean age range was 38 to 40 years. The majority were married (55.6%; 41 192/74 139) and most had a college-level education or higher (40.0%; 29 681/74 244). Most people reported it was their first HIV test (72.9%; 59 181/81 195; Table 2).

Starting from 2021, the quality feedback module covered all voluntary counselling and testing facilities in Guangzhou. Between 2021 and 2023, 3302 posts were anonymously submitted about the quality of voluntary counselling and testing services. In 2023, each top-rated primary health-care facility had an average of 130 visits annually, while lower-ranked facilities averaged only 76 visits per year.

As of 2023, 9622 electronic reports had been forwarded, with 6435 viewed. Additionally, there were 756 online electronic report exchanges.

The total cost of developing and maintaining the WellTest mini-app was 1.5 million Chinese yuan (¥; 207 267 United States dollars, US$), averaging ¥ 300 000 (US$ 41 453) per year and ¥ 8720 (US$ 1205) per primary health-care facility. As the number of covered facilities increases, the investment per facility is expected to decrease. The development and maintenance of the mini-app’s core functionality required approximately five key technical staff members. The cost and implementation leveraged existing government fiscal resources and health-care system personnel, eliminating the need for additional expenditure on platform development and staffing.

## Discussion

After the implementation of WellTest, the majority of visits for voluntary HIV counselling and testing shifted from CDC-based facilities to primary health-care facilities, demonstrating that a digital intervention can redirect such services without requiring additional financial or human resources. Notably, since 2020, despite the impact of coronavirus disease 2019 (COVID-19) on health-care services and an overall decline in client visits, the proportion of clients using voluntary counselling and testing services at primary health-care facilities has increased. Additionally, a higher proportion of newly diagnosed HIV infections have been identified at these facilities, even as the overall number of newly diagnosed HIV infections has decreased. These data suggest a stable HIV epidemic in Guangzhou; however, further confirmatory observations are needed.

Two key factors contributed to the shift in service utilization. First, the development of the WellTest modules followed a bottom-up approach, incorporating core functions designed to meet the needs of both providers and clients. Second, the implementation of WellTest was carefully aligned with the existing structure of the health-care system, leveraging existing counselling and testing resources. This approach aligns with findings from previous research.^36^ The high uptake at primary health-care facilities indicates that WellTest meets the needs of providers, highlighting that the bottom-up approach along with integration into the current health system is important for the successful implementation of digital health interventions in this setting.

Research shows that digital health-care services improve client experience and service efficiency,^37^^,^^38^ particularly encouraging high-risk populations to undergo HIV testing.^39^ Several features of the mini-app contribute to improving services and preventing HIV transmission. First, by expanding clients’ options for voluntary counselling and testing facilities, the likelihood of seeking care is increasing, as it allows them to avoid encounters with acquaintances in their community when visiting the facility. Additionally, providing online counselling and self-test kit collection increases access while reducing the risk of stigmatization. Second, personalized online assistance reaches at-risk populations, facilitating early diagnosis and treatment to prevent HIV transmission. Third, the ability to easily share reports with partners to disclose HIV status can help reduce HIV risk, by encouraging proactive HIV prevention behaviours. Fourth, centralized online assistance addresses disparities in counselling quality across primary health-care facilities. A study in Canada also revealed that centralized information-sharing capabilities can help address disparities in the quality and capacity of primary health-care services.^40^ Finally, the client-centric ratings and feedback feature of the mini-app informs client decisions, drives service improvement and fosters community engagement. This feature aligns with previous studies showing that real-time institutional ratings and feedback provide authentic reviews that influence client choices.^41^^–^^43^ Client feedback also helps voluntary counselling and testing facilities identify issues in staff attitudes, professionalism or institutional environment, enabling targeted improvement.^44^

As the mini-app meets the needs of its users, while also integrating seamlessly into the health system without requiring additional resources, the roll-out has encountered relatively few barriers. However, some implementation challenges should be noted. During the initial roll-out in primary health-care facilities, the increased time and effort required for WellTest training led to complaints from counsellors. To address these complaints, we monitored usage patterns of the mini-app, resolved technical issues and developed features to reduce staff workload. For example, WellTest appointment data were automatically integrated into the National HIV/AIDS Prevention and Control Information System, minimizing manual data entry by counsellors. To manage potential increases in client numbers resulting in higher workloads, facilities could set their own appointment schedules, and counsellors could send online appointment details to clients via WeChat to improve time management.

Our model of integrating digital health interventions with in-person primary health-care services can be replicated in other countries. This approach can increase awareness of primary health-care options, improve disease screening services and address inconsistent service quality. Digital health interventions can help shift HIV testing and other disease screening services to primary health-care settings at a low and manageable cost. To ensure sustainability, these interventions must meet both supply and demand needs, while integrating with the existing health system. Effective implementation requires extensive user engagement to ensure the interventions address their needs, leveraging the current health system structure for information exchange and prioritizing multisectoral coordination. Unified principles for strategies, training standards and operational autonomy at the facility level are also essential. Initial efforts should focus on gaining departmental support to shift from passive promotion to active institutional participation. While adoption and willingness to explore the digital intervention are crucial, long-term sustainability and scalability depend on the intervention’s ability to effectively meet users’ needs.

Our study has some limitations. First, manual data entry at some facilities may have resulted in unrecorded client data in the National HIV/AIDS Prevention and Control Information System at the time of our data extraction, potentially underestimating the true numbers in our analysis. As we used anonymous client data, we were unable to conduct further quantitative surveys or qualitative interviews to explore factors influencing clients’ decisions to seek counselling and testing services at primary health-care facilities. Additionally, the study period included the COVID-19 pandemic, during which various containment measures disrupted the supply and use of voluntary counselling and testing services, potentially affecting the evaluation of WellTest’s implementation.

Some factors contributing to the successful implementation may be unique to China, potentially limiting its replication in other countries. For example, the mini-app module within the WeChat ecosystem simplified app development. Unlike regular apps, mini-apps do not require separate downloading and installation, thereby reducing users’ resistance to adopting the digital intervention.^45^ Furthermore, the initiative was led by the Guangzhou CDC, and lower-level institutions in China are more likely to engage with initiatives promoted by higher-level authorities.

In conclusion, the WellTest mini-app offers low-cost solutions to address underutilization of health services caused by low awareness, limited access to information, the need for certain onsite-only services, stigma and varying service quality. The increased access to voluntary counselling and testing services in primary health-care facilities in Guangzhou highlights the value of digital health interventions in primary health care. Our findings demonstrate the potential for scaling up similar tools to enhance decentralized health care and disease screening in low-resource settings.
